# Surface Loading Proximity Ligation-Induced PCR Technique for Fluorescent Detection of Intact Methicillin-Resistant *Staphylococcus aureus*

**DOI:** 10.4014/jmb.2504.04004

**Published:** 2025-06-23

**Authors:** Tingting Li, Yang Liu, Meijia Zhao, Xue Xin, Shuhong Jin

**Affiliations:** 1 Respiratory Medicine Department, Lequn Campus of the First Hospital of Jilin University, Changchun City, Jilin Province 130000, P.R. China; 2 Nephrology Department, The First Hospital of Jilin University, Changchun City, Jilin Province 130000, P.R. China

**Keywords:** Methicillin-resistant *Staphylococcus aureus*, chronic obstructive pulmonary disease, rolling circle amplification, proximity ligation assays

## Abstract

Methicillin-resistant *Staphylococcus aureus* (MRSA)-induced pneumonia in nursing patients with chronic obstructive pulmonary disease (COPD) necessitates rapid detection, timely intervention, and meticulous clinical management. Thus, the development of sensitive and accurate techniques for MRSA detection holds significant clinical nursing of infectious diseases. In this study, we designed a surface loading proximity ligation assay (PLA) for the precise detection of MRSA in pulmonary infections by simultaneously targeting three characteristic proteins. This assay utilizes three customized probes: the first probe targets protein A on the MRSA surface, the second probe immobilizes on the biological lipid layer, and the third probe identifies PBP2a (a protein responsible for drug resistance of MRSA). The proposed strategy integrates proximity ligation of these three probes with polymerase chain reaction (PCR) to perform “AND” logic-based analysis of the three key MRSA components, enabling sensitive detection of intact MRSA. Taking advantage of the high signal amplification efficiency of PCR and elevated target recognition capability of PLA, the method exhibited a low limit of detection of 2.9 CFU/ml. As a result, the proposed method demonstrated significantly improved accuracy for MRSA detection. We believe this novel integrated strategy could diversify existing bacterial detection approaches and may inspire the development of promising drug candidates in COPD nursing.

## Introduction

Methicillin-resistant *Staphylococcus aureus* (MRSA) has become a major pathogen in nursing-associated infections, particularly among patients with chronic obstructive pulmonary disease (COPD) [1]. COPD, characterized by persistent airflow limitation, increases susceptibility to pulmonary infections due to compromised immune defenses [2]. MRSA resistance is primarily driven by the *mecA* gene, which encodes penicillin-binding protein 2a (PBP2a), conferring resistance to β-lactam antibiotics [3, 4]. As a leading cause of hospital-acquired pneumonia (HAP) and ventilator-associated pneumonia (VAP), MRSA infections are associated with a 30-day mortality rate of 20–40% in nursing [5], significantly higher than that of infections caused by methicillin-sensitive *S. aureus* (MSSA) [6, 7]. Given the complexity and severity of MRSA infections, there is an urgent need for rapid and highly specific diagnostic techniques. Such advancements are essential for guiding timely clinical interventions and improving patient nursing strategies.

Various methods are currently available for the detection of MRSA [10]. Traditional culture-based techniques, while considered the gold standard, are time-consuming, requiring bacterial growth on selective media for 24 to 48 h or longer [8, 9]. These methods are further limited by their dependence on expensive equipment, highly trained personnel [10], and the potential for false-positive results due to contamination [11]. In contrast, PCR-based assays offer faster detection by amplifying specific MRSA DNA sequences [12]. However, the PCR- based methods commonly require complicated primer design, cumbersome equipment for thermal cycles, and tedious nucleic acid extraction steps, limiting its further application. Additionally, immunological techniques, such as enzyme-linked immunosorbent assays (ELISA) [13], can identify MRSA antigens but are often compromised by cross-reactivity with structurally similar bacterial proteins and relatively low sensitivity [14]. In recent years, aptamer-based MRSA detection has emerged as a promising alternative for direct target analysis to conventional methods [15-19]. Aptamers are short, single-stranded nucleic acid molecules that exhibit high binding specificity to target molecules [20-22]. These synthetic ligands can be engineered to recognize unique surface proteins on MRSA, facilitating highly selective detection [23]. However, a major limitation of current aptamer-based systems is their reliance on the identification of a single MRSA surface protein. This single-protein recognition strategy often compromises specificity, as structurally similar proteins from other bacterial species or free proteins in the sample may lead to cross-reactivity and false-positive results [24]. Consequently, these constraints reduce the accuracy and reliability of MRSA detection in complex biological matrices.

Proximity ligation assays (PLA) offer significant potential for improving pathogen detection selectivity by enabling the simultaneous identification of multiple target molecules [25]. Leveraging the unique advantages of PLA, researchers have developed several MRSA detection strategies, achieving substantially enhanced target recognition efficiency [26]. For example, one innovative approach integrates a dual-functional aptamer with CRISPR-Cas12a-mediated rolling circle amplification to enable accurate MRSA identification [24]. However, conventional PLA methods are generally restricted to dual-target detection, limiting their capacity to achieve the high specificity necessary for precise intact MRSA analysis. This limitation highlights the need for novel strategies to expand PLA multiplexing capabilities while maintaining high sensitivity and specificity.

To address these challenges, we developed a triple-functionalized aptamer-based proximity ligation assay coupled with a PCR to induce 3,3’,5,5’-tetramethylbenzidine (TMB) based colorimetric system. This method leverages the synergistic recognition of three target molecules: Protein A (a valuable detection target due to its high specificity and conserved expression across strains, enabling reliable immunological or molecular identification [24]), PBP2a, and the lipid bilayer. By integrating these elements, we established a photocatalysis-based target-induced proximity ligation-mediated PCR technique for the visual detection of MRSA. The functionalized aptamers facilitate protein recognition and convert protein signals into nucleic acid signals, which are then amplified *via* PCR and reported using the color changes of TMB system. This amplification strategy ensures high sensitivity and specificity in MRSA identification. By providing a rapid and accurate MRSA detection method in complex clinical settings, this technique also lays a solid foundation for subsequent clinical decision-making and nursing interventions.

## Experimental Section

### Reagents and Materials

All DNA oligonucleotides (sequences enumerated in Table S1) were manufactured by Shanghai Sangon Biological Engineering Technology and Service Co., Ltd. (China). These probes were developed based on previous research and are suitable for proximity ligation tests and target-specific signal amplification devices. Bacterial strains, such as *S. aureus* and methicillin-resistant *S. aureus*, were procured from Sigma-Aldrich. Penicillin-binding protein 2a (PBP2a) and protein A were acquired from Abcam (USA). The requisite chemicals, comprising 8 strip reagent tubes, Brilliant III Ultra-Fast SYBR Green QPCR premix, and the Agilent Mx3005P QPCR detection equipment, were procured from Agilent Technologies. Concurrently, the SYBR Green I dye, which we obtained, was sourced from Thermo Fisher Scientific. Phosphate-buffered saline (PBS, pH 7.4, catalog number T9181) tablets, provided by Clontech (USA), were integral to the experiment. Enzymes essential for the signal amplification system, including T4 DNA ligase, DNA polymerase, and *Staphylococcus* A lyase, were obtained from Sigma-Aldrich (USA).

### MRSA Detection Analysis

A solution was created for the detection of MRSA. It contained S1/aptamer - protein A (500 nM), S2/aptamer -PBP2a (500 nM), and S3/cholesterol (500 nM). Subsequently, 5 μl of the MRSA sample was added to 15 μl of this solution. The resultant mixture was incubated at 25°C for 45 min. A ligation combination was subsequently prepared. The mixture consisted of 10 μl of 10× T4 DNA ligase buffer (including 10 mM ATP), 2 μl of T4 DNA ligase (0.8 U/ml), and 8 μl of ultrapure water. 20 μl of the ligation mixture was included into the previously incubated sample mixture and thereafter incubated at 25°C for 15 min. Following ligation, 10 μl of the reaction product was combined with 10 μl of 2× PCR master mix containing 1 μM primers. PCR amplification was conducted using the Agilent Mx3005P equipment. The protocol consisted of an initial denaturation at 95°C for 10 min, succeeded by 50 cycles, each comprising 15 sec at 95°C and 30 sec at 60°C. The fluorescence spectra were acquired utilizing a Hitachi F-7000 fluorescence spectrophotometer (Japan).

## Results and Discussion

### Working Mechanism of the Proposed MRSA Tracking Method

**Fig. 1:**
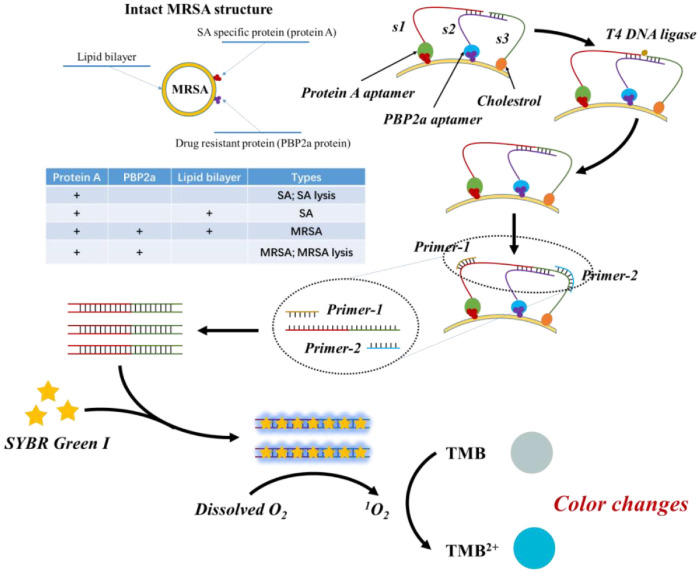
Procedure for identifying MRSA and the mechanism of 3PLA-mediated PCR-induced colorimetric systems.

The working principles of the proposed surface loading proximity ligation based MRSA detection technique are illustrated in Figure 1. Given the critical role of membrane proteins in the biological functions, we employed three specific probes for accurate MRSA identification: S1/aptamer-protein A, S2/aptamer-PBP2a, and S3/cholesterol, designed to target protein A, PBP2a, and the lipid bilayer, respectively. When the S2 probe binds to MRSA, it facilitates hybridization between the 3’ end of S1 and the 5’ end of S3. Subsequently, T4 DNA ligase links S2 and S3 to form a complete DNA sequence, which serves as a template for PCR amplification. Leveraging the high amplification efficiency of PCR, a substantial amount of double-stranded DNA (dsDNA) is generated. Upon binding of SYBR Green I (SGI) to dsDNA, the resulting dsDNA-SGI complex exhibits photocatalytic oxidation activity. Under light exposure, this complex catalyzes the oxidation of the substrate TMB and dissolved oxygen, leading to a significant increase in fluorescence intensity and producing visible orange fluorescence. In summary, this study introduces a novel visual detection method for MRSA, integrating proximity ligation-mediated PCR with a SGI catalyzed signal production system. This approach provides a rapid, sensitive, and highly specific strategy for MRSA detection in clinical settings.

### Identification Efficiency of the Probes to MRSA Surface Protein

**Fig. 2:**
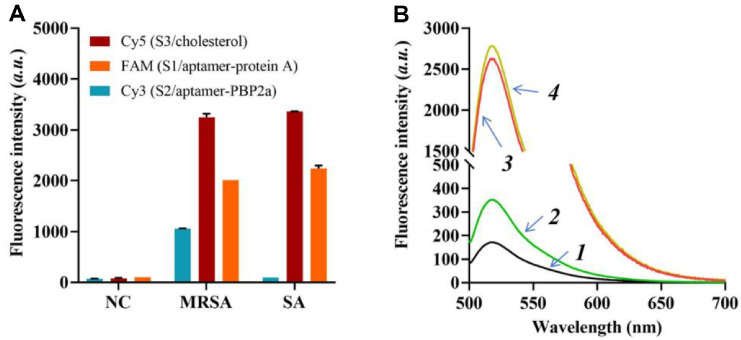
Identification of MRSA using three specifically designed probes. (**A**) Fluorescence intensities observed when the three probes were combined with PBP2a-positive MRSA and PBP2a-negative SA. (**B**) Fluorescence spectra of FAMS3/ cholesterol during the proximity ligation procedure. Line 1: MRSA; Line 2: MRSA combined with FAM and S3/cholesterol; Line 3: MRSA combined with FAM, S3/cholesterol, and BHQ with S1/protein A; Line 4: MRSA combined with FAM, S3/ cholesterol, BHQ with S1/protein A, and S2/aptamer-PBP2a. *P* < 0.05. A *P*-value below 0.05 denotes statistical significance. The data are presented as mean ± standard deviation (**SD**) from 3 independent technical replicates. *P* < 0.01 is the threshold for significance.

MRSA was cultured in a selective medium supplemented with mannitol salt agar for subsequent experimentation. Immunological confirmation of MRSA was performed using a latex agglutination test targeting the PBP2a protein. In the proximity ligation-mediated PCR signal amplification platform, efficient coupling of S1/aptamer-protein A, S2/aptamer-PBP2a, and S3/cholesterol to the MRSA surface was essential. To validate MRSA identification, a fluorescence-based assay was developed using three custom probes labeled with FAM (S1/aptamer-protein A), Cy3 (S2/aptamer-PBP2a), and Cy5 (S3/cholesterol) at their respective termini. Following incubation of isolated MRSA with the fluorescent probes, unbound probes were removed *via* magnetic bead-mediated enrichment and separation. As depicted in Fig. 2A, a significant amplification of Cy3 and Cy5 signals was observed in PBP2a-positive MRSA samples compared to PBP2a-negative *S. aureus* controls (SA). Conversely, within the PBP2a-positive MRSA group, Cy3 and Cy5 signals increased, while the FAM signal decreased.

To further evaluate the feasibility of the triple surface protein recognition mediated PLA system, fluorescence strategies were employed. The 5’ end of the S1 probe was modified with BHQ, whereas the 3’ end of the S3 probe was conjugated with FAM. As shown in Fig. 2B, initial experiments demonstrated a substantial increase in FAM signal intensity upon incubation of the FAM-S3/cholesterol complex with MRSA, followed by removal of unbound S3 (line 2), confirming successful binding. Subsequent addition of BHQ-S1/aptamer-protein A to the mixture did not reduce the FAM signal, indicating an absence of proximity ligation (line 3). However, upon introduction of S2/aptamer-PBP2a, a marked decrease in FAM signal intensity was observed (line 4), suggesting that the S2 probe facilitated close proximity between S1 and S3, enabling effective ligation.

### Assessment and Examination of the Photocatalytic Oxidation Efficacy of the dsDNA - SGI Complex

**Fig. 3:**
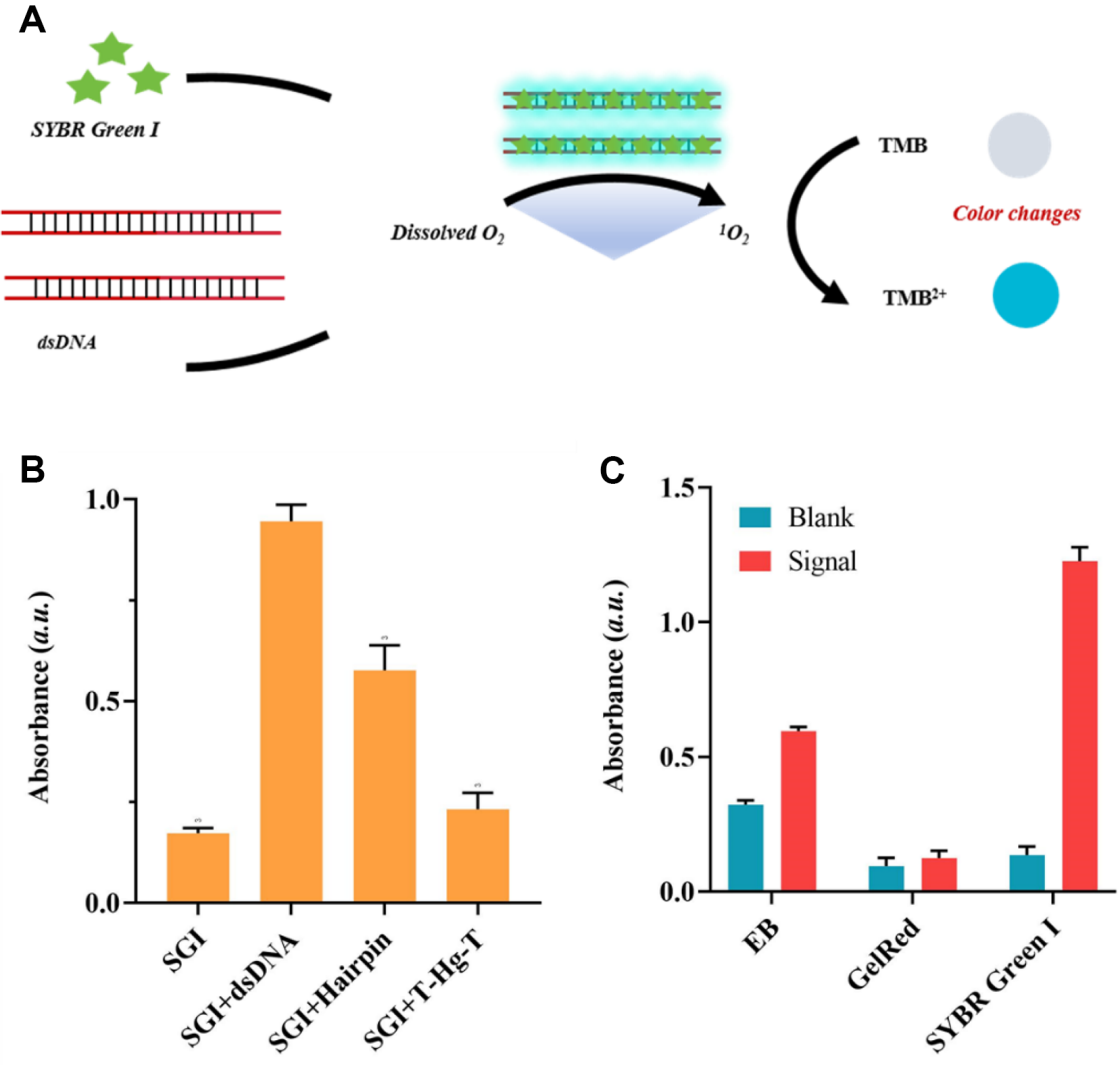
Photocatalytic efficacy of the dsDNA - SGI for TMB. (**A**) Principle of TMB luminescence induced by the dsDNA-SGI complex. (**B**) dsDNA with various configurations showed substantial photocatalytic activity and facilitated the oxidation of TMB. (**C**) In addition to SGI, dsDNA also imparted photocatalytic activity to other intercalating dyes, including EB and GelRed. The data are presented as mean ± standard deviation (**SD**) from 3 independent technical replicates. *P* < 0.01 is the threshold for significance.

Recent experimental researches have conclusively demonstrated that the dsDNA-SGI complex exhibits remarkable photocatalytic oxidation activity. This complex efficiently catalyzes the oxidation of the substrate TMB in the presence of dissolved oxygen under light irradiation. To verify the universality of the dsDNA-SGI system, a fluorescence-based experiment was conducted (Fig. 3A). The experimental conditions were defined as follows: dsDNA concentration was maintained at 100 nM, SGI concentration was set at 3.92 mM, and the GelRed concentration was standardized at 2 mM. Within this experimental setup, three distinct DNA structures—dsDNA, hairpin-structured DNA, and T-Hg^2+^-T mismatched double-stranded DNA—were synthesized and co-incubated with SGI. A pronounced enhancement in SGI fluorescence was observed, indicating successful intercalation of SGI into the dsDNA groove. Upon exposure to blue-light LED irradiation, all three double-stranded DNA variants were found to catalyze TMB oxidation, as evidenced in Fig. 3B. This reaction generated a characteristic absorption peak at 650 nm. Rigorously performed control experiments confirmed that neither ssDNA nor non-intercalated dsDNA exhibited significant photo-oxidase activity. Additionally, the mixture of SGI and ssDNA displayed negligible enzymatic activity. These results collectively underscore that the formation of the dsDNA-SGI complex is essential for photo-oxidase activity. Furthermore, it was experimentally verified that, besides SGI, other intercalating dyes (*e.g.*, ethidium bromide and GelRed) could similarly impart photocatalytic properties to dsDNA upon successful intercalation (Fig. 3C).

### Analytical Performance of the Method

**Fig. 4:**
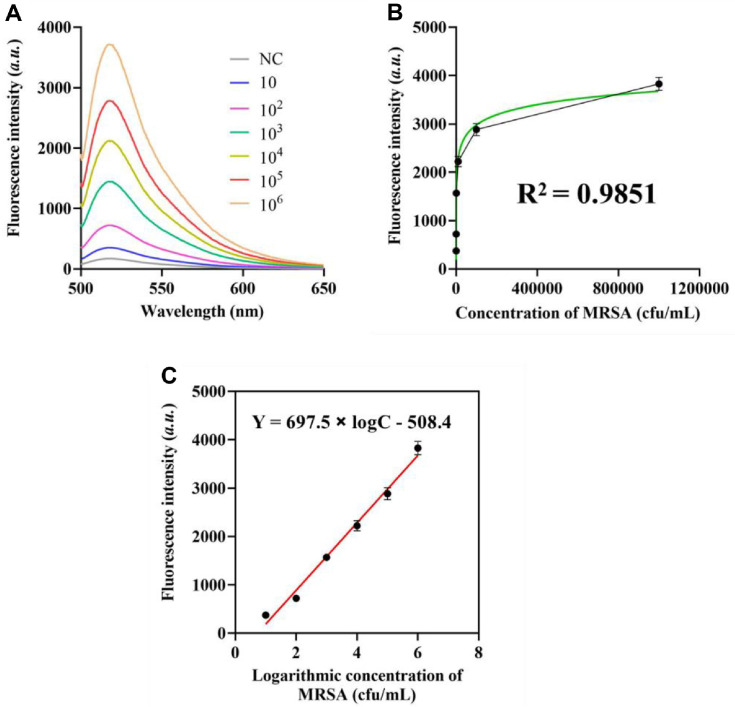
Analytical efficacy of the proposed methodology. (**A**) Fluorescence spectra of the approach for identifying MRSA at varying doses. (**B**) Correlation between peak fluorescence intensity and MRSA concentration. (**C**) Linear correlation between peak fluorescence intensity and logarithmic MRSA concentration. The data are presented as mean ± standard deviation (**SD**) from 3 independent technical replicates. *P* < 0.01 is the threshold for significance.

In this study, we evaluated the efficacy of the proposed method for monitoring MRSA by detecting MRSA isolates from bacterial cultures. The experimental procedure was conducted as follows: First, MRSA colonies were quantified using a standardized colony counting method to ensure precise measurement. Subsequently, the MRSA stock solution was serially diluted to achieve a defined concentration range for sensitivity analysis. The results revealed a strong linear correlation between fluorescence intensity and the logarithmic value of MRSA concentration (Fig. 4A). To further quantify this relationship, a mathematical model was established based on extensive measurement data. The regression analysis demonstrated that fluorescence intensity (Y) was accurately described by the equation: Y = 697.5 × lgC 508.4, where C represents the MRSA concentration. The high coefficient of determination (R² = 0.9851, Fig. 4B and 4C) confirmed the robustness of the linear fit, indicating excellent agreement between the experimental data and the theoretical model. This strong correlation underscores the reliability and precision of the proposed detection method, supporting its potential application in MRSA monitoring. To further validate the sensitivity of the assay, the limit of detection (LOD) was determined to be 2.9 CFU/ml according to the 3δ rule, ensuring the suitability for low-concentration MRSA samples of the method.

### Specificity and Stability Analysis of the Method

**Fig. 5:**
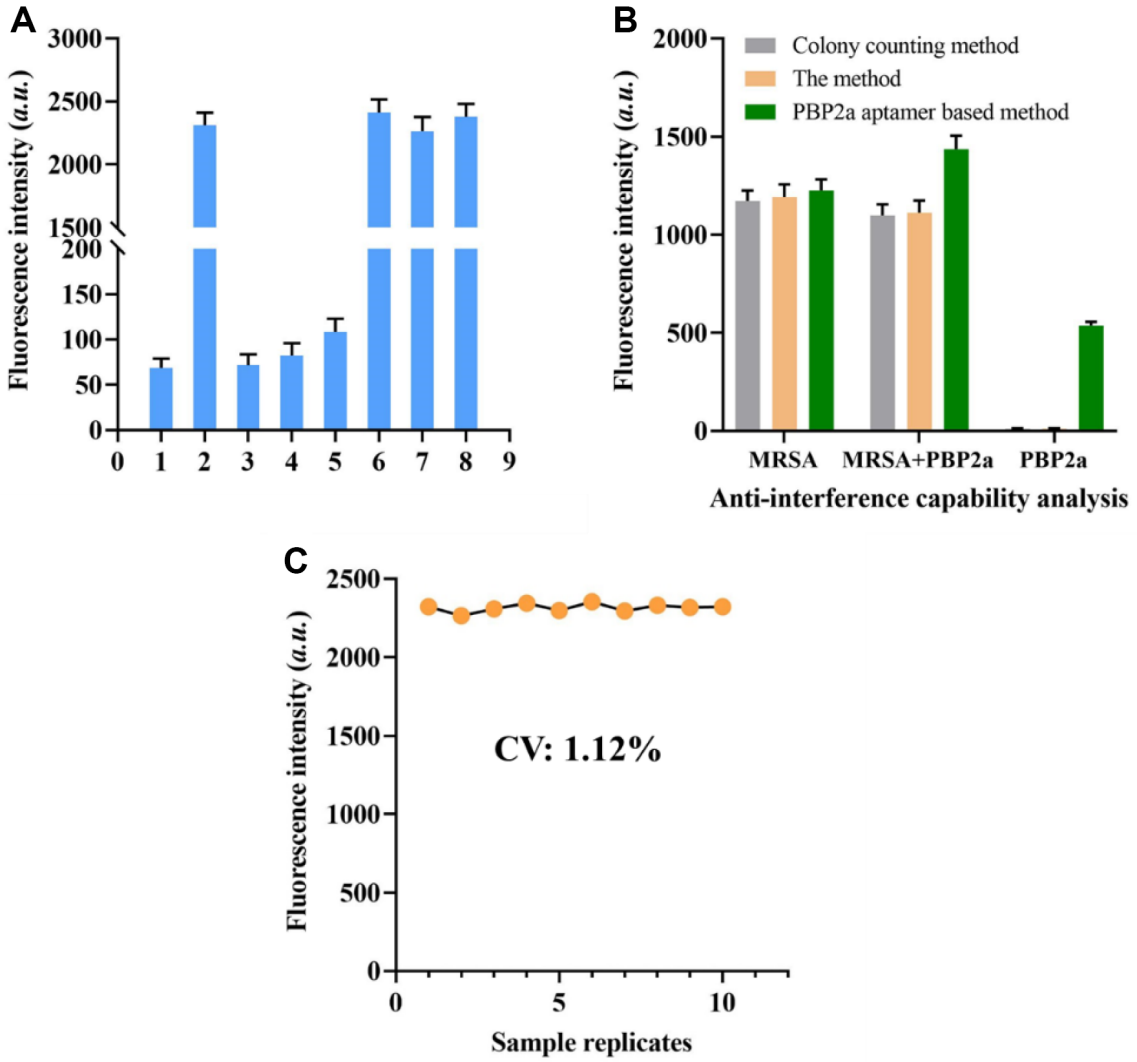
Examination. (**A**) Fluorescence intensity of the approach for identifying MRSA and unbound proteins. Column 1: Negative control; Column 2: FnBPs - positive MRSA; Column 3: FnBPs; Column 4: PBP2a; Column 5: Protein A; Column 6: FnBPs - positive sEV + FnBPs; Column 7: FnBPs - positive MRSA + PBP2a; Column 8: FnBPs - positive MRSA + Protein A. (**B**) Fluorescence intensity of the ELISA method and the proposed method in the detection of MRSA and cell lysis. (**C**) Fluorescence intensity of the technique for identifying 104 CFU/ml MRSA across 10 samples. The data are presented as mean ± standard deviation (SD) from 3 independent technical replicates. *P* < 0.01 is the threshold for significance.

Molecules present on the free surface, such as PBP2a, fibronectin-binding proteins (FnBPs), and protein A, may interfere with the detection efficacy of these methods. To evaluate this effect, we introduced recombinant FnBPs protein into a solution containing 10^6^ CFU/ml of MRSA. As shown in Fig. 5A, the fluorescence enhancement induced by recombinant FnBPs was negligible, indicating that interference from free FnBPs in MRSA samples is minimal. Additionally, interference from free PBP2a, protein A, and cell lysis was found to be insignificant.

Furthermore, selectivity was verified by comparing the proposed method with an established ELISA-based approach. Two parallel experiments were conducted: one using the proposed method and the other employing ELISA. The results demonstrated that the presence of free PBP2a protein had a negligible effect on ELISA or the proposed method. In contrast, the conventional ELISA method produced a significant increase in fluorescence signal for MRSA cell lysis detection (Fig. 5B).

The repeatability of the proposed method was assessed by performing 10 consecutive replicate detections of MRSA. These samples were prepared by diluting the MRSA to the commercial serum solution (mainly the DMEM) to mimic clinical samples. The samples were sequentially processed under identical conditions. The coefficient of variation (CV) for the measured fluorescence intensity was 3.87%, demonstrating high reproducibility (Fig. 5C). This low CV suggests that the method is suitable for clinical applications.

## Conclusion

In this study, we successfully developed an innovative and highly sensitive assay for detecting MRSA in patients with chronic obstructive pulmonary disease. This method employs a three surface protein recognition-mediated proximity ligation assay to simultaneously identify the biological lipid bilayer, protein A, and the distinctive protein PBP2a on the MRSA surface. By enabling multi-surface protein detection, this approach significantly enhances diagnostic precision. Furthermore, we incorporated polymerase chain reaction to achieve efficient signal amplification. Additionally, the novel photocatalytic properties of the dsDNA-SG complex were leveraged for the visual assessment of MRSA. The newly established assay holds substantial clinical relevance, particularly in practical applications. For instance, in nursing-related admission screening, it enables rapid identification of MRSA carriers, allowing for prompt implementation of contact isolation measures. Moreover, in postoperative and intensive care settings, this method facilitates real-time monitoring of high-risk patients, thereby helping to prevent catheter-related infections and surgical site infection outbreaks.

## Supplemental Materials

Supplementary data for this paper are available on-line only at http://jmb.or.kr.
