# Supplementary material for: Surface Loading Proximity Ligation-Induced PCR Technique for Fluorescent Detection of Intact Methicillin-Resistant Staphylococcus aureus
Source: J Microbiol Biotechnol. 2025 Jun 23;35:e2504004. doi: 10.4014/jmb.2504.04004 (PMC12256840; doi:10.4014/jmb.2504.04004)
Supplement: Supplementary file 1 [file jmb-35-e2504004-supple.pdf]

**J. Microbiol. Biotechnol. 2025. 35: e2504004**

**https://doi.org/10.4014/jmb.2504.04004**

# Supplementary Table

**Table S1. Oligonucleotides for nanoparticle-based proximity ligation assay**.

| Title | Sequences (5' to 3') | Modification |
|---|---|---|
| S1 | CAG GTA GTA GTA CGT CTG TTT CAC GAT GAG ACT GGA TGA A | Protein A aptamer |
| S2 | TCA CGG TAG CAT AAG GTG CAA GAT AAT ACT CTC GCA GCA C | PBP2a aptamer |
| S3 | CTA CCG TGA TTC ATC CAG AAA AAA AAA AAA AAA AAA AAA A | Cholesterol |
| Primer-1 | CAG GTA GTA GTA CGT CTG TT | |
| Primer-2 | GTG CTG CGA GAG TAT TAT CT | |

**Supplemented experimental section**

***Bacterial culture***

The culture and isolation protocols for methicillin-resistant Staphylococcus aureus (MRSA) represent standardized microbiological workflows leveraging the pathogen's distinct phenotypic and molecular traits. Selective media such as MRSASelect Agar containing β-lactamase-resistant antibiotics (*e.g.*, cefoxitin or oxacillin) are employed to preferentially propagate MRSA populations while inhibiting susceptible bacterial species. Clinical specimens, including wound exudates and nasal mucosal swabs, undergo pre-enrichment in brain heart infusion (BHI) broth prior to inoculation onto selective agar plates. Incubation at 37°C for 24-48 hours typically yields characteristic opaque, grayish colonies.

***Identification of MRSA bacteria***

Preliminary identification involves Gram staining microscopy confirming Gram-positive cocci in clusters, catalase positivity using 3% hydrogen peroxide, and coagulase testing with rabbit plasma to differentiate *S. aureus* from coagulase-negative staphylococci. Subsequent molecular confirmation relies on either latex agglutination assays targeting PBP2a or PCR amplification of the *mecA* gene. The latter method utilizes validated primers to generate a 310-bp amplicon, offering enhanced specificity compared to phenotypic methods. Antimicrobial susceptibility testing adheres to CLSI guidelines, employing either disk diffusion (Kirby-Bauer) for qualitative assessment of oxacillin resistance (inhibition zone ≤10 mm) or broth microdilution for quantitative minimum inhibitory concentration (MIC) determination. All procedures are conducted under biosafety level 2 (BSL-2) containment, with rigorous quality control measures including reference strain validation (ATCC 43300 for MRSA and ATCC 25923 for MSSA) and media performance verification. This protocol ensures reliable detection of

MRSA isolates, supporting clinical decision-making and infection control strategies in compliance with WS/T 499-2017 and CLSI M100-S33 standards.

**Statistics**

Statistical significances were calculated by Microsoft Excel 2016, and all data were expressed as mean ± standard deviations. The two-tailed Student's t test was used to compare differences between two groups with $P < 0.01$ as a threshold for significance. Technical replicates and sample replicates were performed to improve the statistics.
